# Draft genome of *Streptomyces* sp. AM6-12, a putative novel species for drug discovery isolated from Brazilian Restinga coastal soil

**DOI:** 10.1128/mra.01503-25

**Published:** 2026-03-25

**Authors:** Matheus U. Oliveira, Vicente Almeida Serafim da Silva, Rafael Rocha Rangel, Nildo Alfredo Nhampossa, João Ricardo Vidal Amaral, Daniela Sales Alviano, Cinara Souza da Conceição, Carlos Alberto Xavier Gonçalves, Eamim Daidrê Squizani, Sheila da Silva, Andrew Macrae, Rodrigo Pires do Nascimento

**Affiliations:** 1 Universidade Federal do Rio de Janeiro, Escola de Química, Laboratório de Ecologia e Processos Microbianos 28125, Rio de Janeiro, Brazil; 2 Universidade Federal do Rio de Janeiro, Instituto de Microbiologia, Laboratório de Biotecnologia Sustentável e Bioinformática Microbiana 28125, Rio de Janeiro, Brazil; 3 Universidade Federal do Rio de Janeiro, Instituto de Microbiologia, Laboratório de Estrutura de Superfície de Microrganismos 28125, Rio de Janeiro, Brazil; 4 Instituto SENAI de Inovação em Biossintéticos e Fibras, Rio de Janeiro, Brazil; University of Manitoba, Winnipeg, Manitoba, Canada

**Keywords:** antimicrobial activity, *Streptomyces*, bioactive compounds, secondary metabolites, actinomycetes, Kraken

## Abstract

*Streptomyces* strain AM6-12 was isolated from Restinga de Marambaia soil and is able to completely inhibit the growth of *Escherichia coli, Staphylococcus aureus,* and *Pseudomonas aeruginosa*. Here we report on its genome sequence, with 7,958,853 bp and a 71.89% G+C content.

## ANNOUNCEMENT

Members of genus *Streptomyces* are gram-positive filamentous bacteria known for producing antimicrobial and biotechnologically relevant secondary metabolites (1). *Streptomyces* strain AM6-12 was isolated from Restinga de Marambaia soil (−23.068925°S, −43.960311°W), Rio de Janeiro, Brazil, an ecologically sensitive coastal area. Cultivation and characterization procedures previously performed (2) led to a new species description based on polyphasic analyses, under environmental sampling permit SISBIO/ICMBio N°34203-3. The strain exhibited antagonistic activity against *Staphylococcus aureus* MRSA BMB 9393, *Pseudomonas aeruginosa* ATCC 9027, and *Escherichia coli* ATCC 11229, on Mueller-Hinton agar at 35°C/48 h.

Genomic DNA extraction from spores followed a long-read sequencing-optimized protocol based on mechanical maceration, sodium dodecyl sulfate (SDS)/proteinase K lysis, and alcoholic precipitation (3). DNA was fragmented using G-Tubes (Covaris), and SMRTbell libraries were prepared using the SMRTbell Prep Kit 3.0 and Sequel II Binding Kit 3.2 (Pacific Biosciences), including AMPure PB size selection according to the manufacturer’s instructions. Library concentration was measured using the DNA High Sensitivity Kit (Thermo Fisher Scientific) with a Qubit 4 fluorometer, and fragment size distribution was assessed using the Genomic DNA Kit (Agilent Technologies) with the TapeStation 4200 system. Sequencing was performed using the PacBio Sequel II platform (one SMRT Cell 8M; 2 h of pre-extension, 30 h of data acquisition), generating 60,669 raw reads with an N50 read length of 242,676 bp. Subreads quality control, error correction, and adapter trimming were conducted through the standard PacBio Circular Consensus Sequencing workflow prior to generating HiFi reads as input for genome assembly. Raw read quality was evaluated using FastQC v0.11.9 (4). *De novo* genome assembly was performed using Canu v2.2 (5) with default parameters. Final draft genome assembly was deposited in the NCBI, consisting of four contigs, totaling 7,958,853 bp. Polishing was performed using minimap2 2.28-r1209 and Racon v1.5 with default parameters (6, 7). QUAST v5.0.2 assembly (8), according to the method of Gurevich et al., reported an N50 of 341,342 bp, an L50 of four scaffolds, and a 71.89% G+C content. CheckM v1.1.3 (9) showed 100% completeness and 1.93% contamination. PROKKA v1.14.6 genome annotation (10) predicted 7,126 coding sequences, 46 rRNA genes, 1 tmRNA gene, and 91 tRNA genes.

Kraken2 v2.1.3 taxonomic assignment against the PLUS PFP 2023 database classified 98.37% of 68,114 reads within the genus *Streptomyces* (11). GTDB-Tk v2.4.0 genome-based classification confirmed genus-level placement, with the closest reference genome presenting 92.72% ANI (GCF_030450125.1), corroborating the previous genus-level classification (12). ANI calculations using OrthoANIu v3.8.3 and JSpeciesWS (13) yielded 91.01% identity to *Streptomyces malaysiensis* type strain MUSC 136. These values are below the accepted species threshold (95%–96%), indicating that strain AM6-12 likely represents a novel *Streptomyces* species.

Functional annotation by the Plabase pipeline (14) identified genes associated with nutrient acquisition and plant growth promotion, corroborated by HMMER/PFAM domain analysis. AntiSMASH v8.0 (15) detected multiple biosynthetic gene clusters, including similarities to ε-poly-L-lysine (51%), actinomycin-like clusters (45%), polyoxypeptin (39.7%), and pyrroloformamide (81%). Comparative analysis using clinker v0.0.28 (16) with default parameters demonstrated conserved cluster architecture relative to reference entries in the MIBiG database (17).

*Streptomyces* strain AM6-12 represents a putative novel species within the genus, with genomic features highlighting biotechnological and antimicrobial potential, warranting further investigation of its biosynthetic potential.

## Data Availability

The genome sequence has been deposited at GenBank under accession number JBIQWN000000000.1, BioProject number PRJNA1161424, BioSample number SAMN43777502, and SRA number SRX31707477.
